# Sociodemographic factors, current asthma and lung function in an urban child population

**DOI:** 10.1111/eci.13277

**Published:** 2020-08-11

**Authors:** Junwen Yang‐Huang, Amy van Grieken, Evelien R. van Meel, Huan He, Johan C. de Jongste, Liesbeth Duijts, Hein Raat

**Affiliations:** ^1^ The Generation R Study Group Erasmus Medical Center Rotterdam The Netherlands; ^2^ Department of Public Health Erasmus Medical Center Rotterdam The Netherlands; ^3^ Department of Pediatrics Division of Respiratory Medicine and Allergology Erasmus Medical Center Rotterdam The Netherlands; ^4^ School of Public Administration Southwestern University of Finance and Economics Sichuan China; ^5^ Department of Pediatrics Division of Neonatology Erasmus Medical Center Rotterdam The Netherlands

**Keywords:** asthma, ethnic background, respiratory function tests, socioeconomic status

## Abstract

**Background:**

We aimed to assess which sociodemographic factors are associated with current asthma and indicators of lung function in 10‐year‐old children.

**Methods:**

We analysed data of 5237 children (Mean age: 9.7, SD: 0.3) from the Generation R Study (2012‐2016), a population‐based cohort study in the Netherlands. Indicators of sociodemographic factors included parental educational level, net household income, financial difficulties, parental employment status and child ethnic background. Current asthma (yes/no) was defined as ever doctor‐diagnosed‐asthma combined with wheezing symptoms or asthma‐medication use in the past 12 months. Lung function was measured by spirometry and included forced expiratory volume in 1 second (FEV_1_), forced vital capacity (FVC), FEV_1_/FVC, and forced expiratory flow after exhaling 75% of FVC (FEF_75_). Within‐study sex‐, height‐ and age‐adjusted lung function measurements’ z‐scores were converted.

**Results:**

After adjustment for all sociodemographic factors, an independent association was observed between ethnic background with current asthma and lung function. Compared with children with a Dutch background, children with a nonwestern ethnic background had a higher odds of having current asthma (OR: 1.61, 95% CI: 1.02, 2.53), lower FVC z‐score (−0.25, 95% CI: −0.35, −0.14), higher FEV_1_/FVC z‐score (0.26, 95% CI: 0.14, 0.37) and higher FEF_75%_ z‐score (0.15, 95% CI: 0.04, 0.25).

**Conclusions:**

Among 10‐year‐old children, ethnic background was associated with current asthma and lung function after adjusting for a wide range of sociodemographic factors. No associations were found between socioeconomic status indicators and current asthma. Explanations for these associations such as language barriers, suboptimal care or pathophysiological differences require further investigation.

## 1 INTRODUCTION

Asthma is one of the most common chronic diseases worldwide.^1^ According to phase III (2000‐2003) of the International Study of Asthma and Allergies in Childhood (ISAAC), the global prevalence rate of ever had asthma was 13.8% among 13‐14‐year old children, while the prevalence rate was even higher in western Europe, namely 16.3%.^1^ Childhood asthma is related to school absenteeism, psychosocial problems, life‐threatening exacerbations and impaired quality of life.^2^, ^3^ Previous studies^4^, ^5^, ^6^ suggested that having a low family socioeconomic status (SES) and an ethnic minority background is associated with asthma‐related outcomes. Low family SES has been reported to be associated with more frequent emergency department visits,^7^ more frequent hospitalizations,^8^, ^9^ and higher mortality rates of asthma.^10^ Studies from the United States and the United Kingdom found that children with an ethnic minority backgrounds have higher asthma‐related hospitalizations rates and mortality rates than their peers.^4^, ^11^, ^12^

However, studies regarding the association between such sociodemographic factors and asthma‐related outcomes have showed inconsistent results among children aged 9 and older.^13^ A systematic review showed that the association between SES and asthma varied by the SES indicators used.^14^ Low parental educational level was associated with higher levels of asthma.^15^ Low family income was associated with lower level of asthma.^16^ Furthermore, parental educational level and household income were most frequently used SES indicators.^14^ Associations between other SES indicators (ie parental unemployment and financial difficulties) and asthma can offer a thorough view of socioeconomic inequalities in asthma and yet have not been addressed enough.

Furthermore, measurements of lung function are important for the evaluation of lung development and the presence of asthma but few studies assessed the association between sociodemographic factors and asthma‐related outcomes as well as lung function measurements among children.^17^, ^18^ Mostly children with a Caucasian, African American, South East Asian and North East Asian ethnic background have been studied with regard to lung function measurements. Based on these populations, the reference data for clinical inferences on lung function have been developed.^19^ Ethnic minority groups that are common in western Europe, such as Moroccan, Surinamese and Turkish, are underrepresented in the literature.^20^ No adequate reference data currently exist for the ethnic mix of our study population.

In the present study, we aimed to assess the associations between a wide range of sociodemographic factors (ie parental educational level, net household income, financial difficulties, parental employment status and ethnic background) with current asthma (ever doctor‐diagnosed‐asthma combined with wheezing symptoms or asthma‐medication use in the past 12 months) among 10‐year‐old children. Secondly, we calculated within‐study sex‐, height‐ and age‐adjusted lung function measurements’ z‐scores. We explored the associations between family SES, ethnic background and the lung function measurements’ z‐scores.

## 2 MATERIALS AND METHODS

### 2.1 Study design

This study was embedded in the Generation R Study, a population‐based prospective cohort study from early foetal life onwards in Rotterdam, the Netherlands. A detailed description of the study design and participant inclusion procedure has been published previously.^21^ Consent for postnatal follow‐up was available for 7393 children. Children with missing data on asthma or lung function (n = 1609), and on all sociodemographic factors (n = 111) were excluded. To avoid clustering of data, second (n = 427) and third children (n = 9) of the same mother were excluded, leaving a study population of 5237 participants. The study was conducted in accordance with the guidelines proposed in the Declaration of Helsinki. The Medical Ethics Committee of the Erasmus Medical Center, Rotterdam, approved the study. Written informed consent was obtained from all participants.

### 2.2 Sociodemographic factors

Sociodemographic factors included maternal and paternal educational level, net household income, financial difficulties, maternal and paternal employment status, and child ethnic background. Maternal and paternal educational level were obtained by questionnaire when the child was 6 years old and categorized as follows: low (no education, primary school, lower vocational training, intermediate general school, or three years or less general secondary school), mid‐low (more than three years general secondary school, intermediate vocational training, or first year of higher vocational training), mid‐high (higher vocational training) and high (university or PhD degree).^22^ Self‐reported net household income (<€2000/month, €2000‐€3200/month, >€3200/month)^23^ and maternal and paternal employment status (no paid job and paid job) were obtained by questionnaire at child age 6 years. Financial difficulties (yes, no) were defined as difficulties in paying rent, electricity bills, food and suchlike during the past year, assessed by a questionnaire at child age 2 years. Child ethnic background (Dutch, other western and nonwestern) was based on the country of birth of the parents, which was assessed by questionnaires when the child was 6 years old. If one of the parents was born outside the Netherlands, this country of birth determined the ethnic background of the child. If both parents were born outside the Netherlands, the country of birth of the mother determined the ethnic background.^24^

### 2.3 Current asthma and lung function

Current asthma at the age of 10 years (yes or no) was defined as ever diagnosis of asthma (yes or no), with either wheezing (yes or no) or medication use (yes or no) in the past 12 months. Information on whether the child ever received a diagnosis of asthma and whether the child suffered from wheezing in the past 12 months was obtained by questionnaire using adapted items of the ISAAC core questionnaires.^25^ Information on asthma‐related medication use in the past 12 months was obtained during the child's visit at the research centre at age 10 years. During the visit, lung function was measured by spirometry according to the American Thoracic Society and European Respiratory Society guidelines^26^ and included Forced Expiratory Volume in the first second (FEV_1_), Forced Vital Capacity (FVC), FEV_1_/FVC and Forced Expiratory Flow after exhaling 75% of FVC (FEF_75_). Lung function measurements were converted into study specific sex‐, height‐ and age‐adjusted z‐scores using multiple regression analysis. The general form of the equation was as follows: *Y* = *a* + *b* × height + *c* × age for boys and girls separately. Each value of lung function measurement, height or age was log transformed. The goodness of fit was judged from inspection of normal Q‐Q plots.

### 2.4 Statistical analyses

The associations between sociodemographic factors and current asthma were assessed by logistic regression models adjusting for confounders: maternal age, marital status, parity, child gender and exact age at measurement. The associations between sociodemographic factors and lung function measurements were assessed by linear regression models adjusting for maternal age, marital status and parity. The first set of models included each indicator of sociodemographic factors separately, adjusted with confounders (ie basic models). The second set of models included all indicators of sociodemographic factors (ie full models) to assess the independent effects of each sociodemographic factor. Interaction effects between ethnic background and each SES indicator were assessed with UNIANOVA. Bonferroni correction was applied for multiple testing (*P* = .10/30 = .003).^27^ Collinearity analysis using linear regression yielded acceptable collinearity (VIF <3) between SES indicators; therefore, these variables were included simultaneously in the full models. Effect estimates (ORs and z‐score difference) and their 95% confidence intervals (CIs) were reported. Statistical analyses were performed using IBM SPSS statistics for Windows, version 24.0. Armonk, NY: IBM Corp.

### 2.5 Nonresponse analyses

Sociodemographic factors of children with missing data on current asthma and lung function measurements (n = 1609) were compared with those of children without missing data (n = 5237) using chi‐square tests. Data were more often missing for children from parents with a low maternal or paternal educational level, a low household income, a family with financial difficulties, a mother or father without a paid job, or from nonwestern ethnic background (all *P* < .05).

### 2.6 Sensitivity analyses

Sensitivity analyses were performed using specific groups of nonwestern population (ie Moroccan, Turkish and Surinamese and other nonwestern) in the full model to explore the associations between ethnic background and asthma‐related outcomes (see Appendix Table A2). Also, we explored the associations between each SES indicator separately and asthma‐related outcomes adjusting for ethnic background (see Appendix Table A3). Possible residual confounding was explored by additionally adjusted for a wide range of other potential confounders (ie child's birth weight, gestational age, ever eczema at age 9 years, respiratory tract infections, maternal age at enrolment, marital status, parity, maternal smoking during pregnancy, ever breastfeeding, pets exposure at home, daycare attendance and maternal BMI before pregnancy) in the full model (see Appendix Table A4). Stratified analyses were performed in the association between ethnic background and lung function with or without current asthma (see Appendices Table A5 and A6).

## 3 RESULTS

### 3.1 Participant characteristics

Table 1 summarizes the characteristics of the participants stratified by current asthma (5.9%) or no current asthma (94.1%) at age 10 years (mean: 9.7, SD: 0.3). Children with current asthma were more likely to have a mother with low educational level, and belong to a household with a net income of less than €2000/month (both *P* < .05). Compared with children without current asthma, children with current asthma more often were male, with a nonwestern ethnic background, had a lower FEV_1_, lower FEV_1_/FVC and lower FEF_75%_ (*P* < .05).

**Table 1:** Characteristics of children and their mothers (N = 5237)

	Total	Current Asthma	No current asthma	P‐value^a^
	N = 5237	N = 259 (5.9)	N = 4161 (94.1)	
Parental characteristics				
Maternal education				
Low	395 (9.8)	34 (15.2)	361 (9.5)	.02
Mid‐low	1183 (29.4)	71 (31.7)	1112 (29.2)	
Mid‐high	1167 (29.0)	56 (25.0)	1111 (29.2)	
High	1281 (31.8)	63 (28.1)	1218 (32.0)	
Paternal education				
Low	506 (13.7)	40 (20.1)	466 (13.3)	.06
Mid‐low	961 (26.0)	46 (23.1)	915 (26.2)	
Mid‐high	891 (24.1)	44 (22.1)	847 (24.2)	
High	1340 (36.2)	69 (34.7)	1271 (36.3)	
Net household income				
Less than €2000/month	738 (19.3)	55 (25.9)	683 (18.9)	.01
€2000/month‐€3200/month	1002 (26.2)	63 (29.7)	939 (26.0)	
More than €3200/month	2088 (54.5)	94 (44.3)	1994 (55.1)	
Financial difficulties (Yes)	612 (18.5)	41 (23.4)	571 (18.2)	.09
Maternal unemployment	802 (20.9)	47 (22.5)	755 (20.8)	.57
Paternal unemployment	176 (4.9)	12 (6.6)	164 (4.8)	.28
Children's characteristics				
Child ethnic background				
Dutch	2826 (64.1)	141 (54.4)	2685 (64.7)	<.001
Other western	387 (8.8)	14 (5.4)	373 (9.0)	
Nonwestern	1197 (27.1)	104 (40.2)	1093 (26.3)	
Female sex	2216 (50.1)	95 (36.7)	2121 (51.0)	<.001
FEV_1_, mean (SD), L	2.01 (0.29)	1.97 (0.30)	2.01 (0.29)	.05
FEV_1_ z‐score, mean (SD)	0.03 (0.97)	−0.14 (1.04)	0.04 (0.97)	.02
FVC, mean (SD), L	2.33 (0.36)	2.36 (0.36)	2.33 (0.36)	.14
FVC z‐score, mean (SD)	0.02 (0.97)	0.14 (1.08)	0.02 (0.97)	.11
FEV_1_/FVC, mean (SD), %	86.70 (5.71)	83.90 (6.65)	86.87 (5.60)	<.001
FEV_1_/FVC z‐score, mean (SD)	0.01 (0.98)	−0.43 (1.16)	0.04 (0.97)	<.001
FEF_75_, mean (SD), L/s	1.14 (0.34)	1.02 (0.34)	1.15 (0.34)	<.001
FEF_75_ z‐score, mean (SD)	0.02 (0.98)	−0.34 (0.95)	0.04 (0.98)	<.001

^a^ Chi‐square tests were used for categorical variables and independent t tests were used for continuous variables.

### 3.2 Sociodemographic factors and current asthma

Children of low‐educated mothers (OR: 1.81, 95% CI: 1.13, 2.91) had higher odds of having current asthma compared with children of high‐educated mothers (Basic models, Table 2). Children from low‐income households (OR: 1.71, 95% CI: 1.15, 2.54) and middle‐income households (OR: 1.43, 95% CI: 1.01, 2.03) had higher odds of having current asthma compared with children living in high‐income households. Children with a nonwestern ethnic background (OR: 1.64, 95% CI: 1.22, 2.20) had higher odds of having current asthma compared with children with a Dutch background.

**Table 2:** Associations of sociodemographic factors with current asthma and lung function at 10 years of age (basic models)

	OR (95% CI)	z‐score difference (95% CI)			
	Current asthma^a^	FEV_1_ ^b^	FVC^b^	FEV_1_/FVC^b^	FEF_75_ ^b^
	n = 4420	n = 4641	n = 4641	n = 4641	n = 4641
Maternal educational level					
High	Reference	Reference	Reference	Reference	Reference
Mid‐high	0.94 (0.64, 1.38)	−0.03 (−0.11, 0.05)	0.001 (−0.08, 0.08)	−0.05 (−0.13, 0.03)	−0.02 (−0.10, 0.06)
Mid‐low	1.19 (0.81, 1.74)	−0.06 (−0.14, 0.02)	−0.07 (−0.16, 0.01)	0.02 (−0.07, 0.10)	0.05 (−0.04, 0.13)
Low	**1.81 (1.13, 2.91)**	0.02 (−0.09, 0.13)	−0.02 (−0.13, 0.09)	0.08 (−0.03, 0.20)	0.10 (−0.01, 0.22)
Paternal educational level					
High	Reference	Reference	Reference	Reference	Reference
Mid‐high	0.86 (0.57, 1.30)	−0.04 (−0.12, 0.05)	−0.04 (−0.13, 0.04)	0.01 (−0.08, 0.09)	0.03 (−0.06, 0.11)
Mid‐low	0.89 (0.59, 1.33)	−0.04 (−0.12, 0.04)	**−0.09 (−0.17, −0.003)**	0.07 (−0.01, 0.16)	0.07 (−0.02, 0.15)
Low	1.27 (0.81, 2.00)	**−0.11 (−0.21, −0.01)**	**−0.16 (−0.26, −0.06)**	0.08 (−0.03, 0.18)	0.05 (−0.06, 0.15)
Net household income					
More than €3200/month	Reference	Reference	Reference	Reference	Reference
€2000‐€3200/month	**1.43 (1.01, 2.03)**	−0.001 (−0.08, 0.08)	−0.04 (−0.11, 0.04)	0.06 (−0.02, 0.13)	0.05 (−0.03, 0.12)
Less than €2000/month	**1.71 (1.15, 2.54)**	−0.09 (−0.19, 0.01)	**−0.14 (−0.24, −0.05)**	0.08 (−0.02, 0.18)	0.05 (−0.05, 0.15)
Financial difficulties					
No	Reference	Reference	Reference	Reference	Reference
Yes	1.27 (0.86, 1.87)	0.06 (−0.03, 0.16)	−0.04 (−0.10, 0.09)	**0.12 (0.03, 0.21)**	**0.10 (0.01, 0.19)**
Paternal unemployment					
Paid job	Reference	Reference	Reference	Reference	Reference
No paid job	1.33 (0.70, 2.51)	−0.04 (−0.18, 0.10)	−0.02 (−0.16, 0.12)	−0.03 (−0.17, 0.11)	−0.04 (−0.18, 0.10)
Maternal unemployment					
Paid job	Reference	Reference	Reference	Reference	Reference
No paid job	1.07 (0.75, 1.52)	0.06 (−0.01, 0.14)	**0.08 (0.01, 0.16)**	−0.04 (−0.11, 0.04)	−0.01 (−0.08, 0.07)
Ethnic background^c^					
Dutch	Reference	Reference	Reference	Reference	Reference
Other western	0.72 (0.40, 1.28)	**0.13 (0.02, 0.23)**	**0.11 (0.002, 0.22)**	0.04 (−0.06, 0.15)	0.08 (−0.03, 0.19)
Nonwestern	**1.64 (1.22, 2.20)**	**−0.18 (−0.25, −0.11)**	**−0.28 (−0.35, −0.21)**	**0.18 (0.11, 0.25)**	0.05 (−0.03, 0.12)

Note

Bold print indicates statistical significance. Each sociodemographic factor was added to the model separately.

^a^ Models were adjusted for maternal age at enrolment, marital status, parity, child's gender and exact age at measurement.

^b^ Models were adjusted for maternal age at enrolment, marital status and parity.

^c^ Other western: Oceanian, European, American and Japanese. Nonwestern: Antillean, Cape Verdean, Moroccan, Surinamese, Turkish, Kurdish and others.

After adjustment for all indicators in the model, an independent association was observed between ethnic background and current asthma only (Full models, Table 3). Children with a nonwestern ethnic background (OR: 1.61, 95% CI: 1.02, 2.53) had a higher odds of having current asthma compared with children with a Dutch background.

**Table 3:** Associations of sociodemographic factors with current asthma and lung function at 10 years of age (full models^a^)

	OR (95% CI)	z‐score difference (95% CI)			
	Current asthma^a^	FEV_1_ ^b^	FVC^b^	FEV_1_/FVC^b^	FEF_75_ ^b^
	n = 4420	n = 4641	n = 4641	n = 4641	n = 4641
Maternal educational level					
High	Reference	Reference	Reference	Reference	Reference
Mid‐high	0.95 (0.59, 1.53)	0.00 (−0.10, 0.10)	0.04 (−0.06, 0.14)	−0.06 (−0.16, 0.04)	−0.02 (−0.12, 0.08)
Mid‐low	1.18 (0.69, 2.04)	−0.02 (−0.14, 0.09)	0.02 (−0.09, 0.14)	−0.09 (−0.21, 0.03)	−0.001 (−0.12, 0.12)
Low	1.25 (0.53, 2.96)	0.16 (−0.04, 0.36)	0.14 (−0.06, 0.35)	−0.02 (−0.23, 0.19)	0.11 (−0.09, 0.31)
Paternal educational level					
High	Reference	Reference	Reference	Reference	Reference
Mid‐high	0.65 (0.39, 1.08)	−0.03 (−0.13, 0.07)	−0.05 (−0.15, 0.05)	0.03 (−0.07, 0.13)	0.05 (−0.05, 0.15)
Mid‐low	0.61 (0.35, 1.07)	−0.05 (−0.16, 0.06)	−0.08 (−0.19, 0.03)	0.01 (−0.11, 0.13)	0.03 (−0.08, 0.14)
Low	1.07 (0.55, 2.08)	−0.03 (−0.18, 0.13)	−0.04 (−0.20, 0.12)	0.03 (−0.13, 0.20)	0.01 (−0.15, 0.17)
Net household income					
More than €3200/month	Reference	Reference	Reference	Reference	Reference
€2000‐€3200/month	1.17 (0.72, 1.91)	0.01 (−0.09, 0.11)	−0.01 (−0.11, 0.09)	0.04 (−0.06, 0.15)	0.01 (−0.10, 0.11)
Less than €2000/month	1.43 (0.66, 3.09)	−0.12 (−0.30, 0.05)	−0.16 (−0.34, 0.02)	0.09 (−0.10, 0.28)	0.01 (−0.17, 0.19)
Financial difficulties					
No	Reference	Reference	Reference	Reference	Reference
Yes	0.89 (0.53, 1.50)	0.08 (−0.03, 0.19)	0.02 (−0.09, 0.13)	**0.12 (0.01, 0.24)**	0.08 (−0.04, 0.19)
Paternal unemployment					
Paid job	Reference	Reference	Reference	Reference	Reference
No paid job	0.50 (0.17, 1.46)	−0.08 (−0.27, 0.12)	−0.01 (−0.20, 0.19)	−0.11 (−0.31, 0.09)	−0.14 (−0.33, 0.06)
Maternal unemployment					
Paid job	Reference	Reference	Reference	Reference	Reference
No paid job	0.88 (0.52, 1.47)	0.08 (−0.03, 0.19)	**0.14 (0.03, 0.25)**	−0.09 (−0.20, 0.02)	−0.06 (−0.17, 0.05)
Ethnic background^c^					
Dutch	Reference	Reference	Reference	Reference	Reference
Other western	0.94 (0.48, 1.86)	0.13 (−0.01, 0.26)	0.12 (−0.01, 0.26)	0.01 (−0.12, 0.15)	0.09 (−0.04, 0.23)
Nonwestern	**1.61 (1.02, 2.53)**	−0.10 (−0.20, 0.01)	**−0.25 (−0.35, −0.14)**	**0.26 (0.14 0.37)**	**0.15 (0.04, 0.25)**

Note

Bold print indicates statistical significance.

All sociodemographic factors were added to the model.

^a^ Models were adjusted for maternal age at enrolment, marital status, parity, child's gender and exact age at measurement.

^b^ Models were adjusted for maternal age at enrolment, marital status and parity.

^c^ Other western: Oceanian, European, American and Japanese. Nonwestern: Antillean, Cape Verdean, Moroccan, Surinamese, Turkish, Kurdish and others.

### 3.3 Sociodemographic factors and lung function

When compared with high paternal educational level, low paternal educational level was associated with lower FEV_1_ (z‐score difference: −0.11, 95% CI: −0.21, −0.01) and lower FVC (z‐score difference: −0.16, 95% CI: −0.26, −0.06) (Basic models, Table 2). Compared with children from a household income more than €3200/month, low household income (less than €2000/month) was associated with lower FVC (z‐score difference: −0.14, 95% CI: −0.24, −0.05). Financial difficulties were associated with higher FEV_1_/FVC (z‐score difference: 0.12, 95% CI: 0.03, 0.21) and higher FEF_75_ (z‐score difference: 0.10, 95%CI: 0.01, 0.19). Maternal unemployment was associated with higher FVC (z‐score difference: 0.08, 95% CI: 0.01, 0.16). Table 2 shows differences between ethnic subgroups and lung function measurements. Compared to children with a Dutch background, a nonwestern ethnic background was associated with lower FEV_1_ (z‐score difference: −0.18, 95% CI: −0.25, −0.11) and lower FVC (z‐score difference: −0.28, 95% CI: −0.35, −0.21). The difference in FVC exceeded that of FEV_1_, so that FEV_1_/FVC was higher in children with a nonwestern ethnic background (z‐score difference: 0.18, 95% CI: 0.11, 0.25).

After adjustment for all sociodemographic factors in the model, independent associations were observed for ethnic background with lung function measurements (Full models, Table 3). Compared with children with a Dutch background, a nonwestern ethnic background was associated with lower FVC (z‐score difference: −0.25, 95% CI: −0.35, −0.14), higher FEV_1_/FVC (z‐score difference: 0.26, 95% CI: 0.14, 0.37) and higher FEF_75_ (z‐score difference: 0.15, 95% CI: 0.04, 0.25). Also, maternal unemployment was associated with higher FVC (z‐score difference: 0.14, 95% CI: 0.03, 0.25). Children from a family with financial difficulties were more likely to have higher FEV_1_/FVC (z‐score difference: 0.12, 95% CI: 0.01, 0.24).

### 3.4 Interaction effects

Apart from an interaction effect between ethnic background and maternal unemployment, no statistically significant interaction effects were found. All P‐values of the interaction effect analyses are presented in Appendix Table A1.

### 3.5 Sensitivity analyses

Appendix Table A2 shows that children with a Surinamese ethnic background had higher odds (OR: 2.52, 95% CI: 1.29, 4.90) of having current asthma compared with children with a Dutch background. Results from Appendix Table A3 are comparable to the main analyses, although effect estimates (z‐score difference) were larger. No significant association was found between ethnic background and current asthma after adjusting for extra potential confounders (Appendix Table A4). Stratified analyses showed that among children without current asthma, results were comparable to the main analyses (Appendix Table A5). Among children with current asthma, no association was found between ethnic background and lung function (Appendix Table A6).

## 4 DISCUSSION

This study contributes to the knowledge regarding sociodemographic risk factors for asthma‐related outcomes in a sample of European children with diverse ethnic background. After adjustment for all sociodemographic factors, maternal unemployment was associated with higher FVC and financial difficulties with higher FEV_1_/FVC. Children with a nonwestern ethnic background were significantly more likely to have current asthma, lower FVC, higher FEV_1_/FVC and higher FEF_75_.

With regard to asthma, a systematic review reported that among children aged 9 and younger, lower family SES, including lower parent occupation and higher poverty status, are associated with asthma.^13^ However, among children aged 9 years and older, these associations were not apparent.^13^ Our study supports this finding, as we also did not observe an inverse association between family SES and asthma at school‐age after correcting for other sociodemographic factors including a wide range of family SES indicators. A possible explanation might be that when children grow older, they tend to spend more time at school or outside with their friends instead of staying at home. Thus, the impact of poor housing conditions, which children from low SES families tend to be exposed to, may be larger in early childhood than at later age.^13^, ^28^

Our findings regarding differences in asthma prevalence according to ethnic background correspond to earlier studies showing higher risk of asthma among preschool children, school‐aged children, and adolescents from ethnic minority groups.^4^, ^29^, ^30^ Our study adds to the evidence on association between ethnic background and asthma by showing such differences remain after adjustment for a wide range of family SES indicators at 10‐years of age. The higher risk of asthma among nonwestern children is not fully explained by low‐income or low educational level. Previous studies showed that differences in asthma between subgroups with different ethnic backgrounds were independent of indicators of SES and could only partly be explained by bad housing (eg houses infested with rodents and lacking sufficient heat) and neighbourhood conditions (eg little/no social cohesion and boarded‐up buildings nearby).^31^ In our study, when we evaluated the nonwestern population and added them to the full model as specific groups, only children with a Surinamese ethnic background had higher odds of having current asthma compared to children with Dutch background (Appendix Table A2). Interpretation of these results should be done with caution because of a lack of statistical power. Future studies on differences in asthma among ethnic subgroups are needed to also provide insight in language barriers in care, suboptimal care or pathophysiological differences, especially in western Europe.

Children with a nonwestern ethnic background had lower FEV_1_ and FVC than their Dutch peers. These findings are in line with previous studies reporting differences in lung function between subgroups with different ethnic backgrounds in age groups varying from the preschool period until adolescence.^19^, ^32^ A study in United Kingdom showed that Black African/Caribbean and South Asian children were found to have lower FEV_1_ and FVC than white children.^32^ Another study in the United States has reported that African American children were taller but had lower FEV_1_ and FVC than white children.^18^ In our study, the relatively low in FEV_1_ and FVC in children with a nonwestern ethnic background did not seem to reflect on airway obstruction, as their FEV_1_/FVC ratios and end‐expiratory flows were not low, but were slightly higher. This may be due to reduced lung and airway size rather than obstruction. However, such smaller airways may represent a risk factor for asthma symptoms in children,^33^ which provides a possible explanation of differences in asthma between subgroups with different ethnic backgrounds. Another explanation could be the difference in developmental age of puberty between populations.^34^, ^35^ In childhood, FVC outgrows FEV_1_, leading to falls in FEV_1_/FVC; these trends are reversed in adolescence. FEV_1_/FVC ratios are higher in the children shorter for their age.^35^ Furthermore, stratified analyses showed that no association was found between ethnic background and lung function among children with current asthma. One possible explanation could be that medication was used to relieve the symptoms and thus improve the lung function among children with current asthma. Cautious interpretation of these results is needed because of the small sample size in the subgroup. We suggest that clinical practitioners pay attention to potential differential development of lung function among children with a migration background.

Independent association was observed for maternal unemployment with higher FVC after adjustment for all sociodemographic factors. This was an unexpected finding and not consistent with results of other lung function measurements. Further research is warranted to confirm the association between maternal employment status and child lung function.

### 4.1 Methodological considerations

A strength of this diverse urban population‐based study is the large number of subjects being studied with detailed and prospectively measured information on a wide range of indicators of family SES and specific lung function measurements.

Some limitations of the study have to be considered in the interpretation of the results. Child's ethnic background was defined according to the standard methods used in the Netherlands.^24^ This definition implies that third generation immigrants were labelled as Dutch and were hence not distinguished. This may lead to reduction in the contrast between Dutch and other ethnic backgrounds, and the effect sizes then would be relatively smaller. Information on ever diagnosis of asthma and wheezing in the past 12 months was obtained by parental report using the questions from the ISAAC, a validated instrument in epidemiologic studies.^36^ However, misclassification attributable to low parental awareness might be present.

Table 3 showed the associations between sociodemographic background and five asthma‐related outcomes. However, with seven SES indicators together in each of the five models, there may be concerns for overlap between these factors. Although there appeared to be no multicollinearity (see method section), we performed sensitivity analyses to explore the associations between each SES indicator separately with asthma‐related outcomes adjusting ethnic background in the models. Similar results were found for the associations between SES indicators and asthma‐related outcomes (Appendix Table A3). Apart from maternal unemployment, no associations were found between SES indicators and asthma‐related outcomes after adjusting for ethnic background.

Another related argument concerns the possible residual confounding when assessing sociodemographic factors with asthma and lung function measurements. When we additionally adjusted for a wide range of other potential confounders, no significant association was found between ethnic background and current asthma (Appendix Table A4). The associations between ethnic background and FVC and FEV_1_/FVC remained. However, these additional variables in the model may also be considered as mediators, explaining the associations between sociodemographic factors and asthma.^37^, ^38^ Therefore, they were excluded in the main analyses. Future studies should explore specific pathways related to the differences in asthma‐related outcomes between subgroups with different ethnic backgrounds.

## 5 CONCLUSIONS

This study showed that after adjusting for a wide range of sociodemographic factors, children with a nonwestern ethnic background were more likely to have higher risk of current asthma, smaller lung volumes (FVC), but higher FEV_1_/FVC and mid‐expiratory flows (FEF_75_) than children with a majority ethnic background. No associations were found between SES indicators and current asthma. Explanations for these associations such as language barriers, suboptimal care or pathophysiological differences require further investigation in longitudinal studies. In the meantime, physicians, nurses and other healthcare professionals should be aware of the relatively high prevalence of asthma among children with a migration background in European cities.

## CONFLICT OF INTEREST

All authors declare that they have no conflict of interest.

## AUTHORS' CONTRIBUTIONS

JYH, AvG and HR conceptualized and designed the study. JYH performed the statistical analyses. JYH drafted the manuscript. AvG and HR supervised the data analyses. ERvM and LD contributed to methodology considerations. AvG, ERvM, HH, JCdJ, LD and HR reviewed the manuscript for important intellectual content.

## Supporting information

Supplementary Material

## Appendix A

**Table A1:** P‐values for interaction effects between ethnic background and each socioeconomic status variables on current asthma and lung function measurement

Items	Current asthma	FEV_1_	FVC	FEV_1_/FVC	FEF_75_
	P‐value	P‐value	P‐value	P‐value	P‐value
Ethnic background × maternal educational level	.980	.120	.066	.626	.903
Ethnic background × paternal educational level	.924	.085	.091	.572	.410
Ethnic background × net household income	.596	.471	.514	.089	.553
Ethnic background × financial difficulties	.783	.354	.186	.762	.899
Ethnic background × paternal unemployment	.958	.483	.920	.332	.492
Ethnic background × maternal unemployment	.905	.011	**.002**	.456	.515

Note

Significant P‐values in bold. After applying Bonferroni correction for multiple testing (*P* = .10/30 = 0.003), except interaction effect between ethnic background and maternal unemployment, no statistically significant interaction effect was found.

**Table A2:** Associations of sociodemographic factors with current asthma and lung function at 10 years of age (full models)

	OR (95% CI)	z Score change (95% CI)			
	Current asthma^a^	FEV_1_ ^b^	FVC^b^	FEV_1_/FVC^b^	FEF_75_ ^b^
	n = 4420	n = 4641	n = 4641	n = 4641	n = 4641
Maternal educational level					
High	Reference	Reference	Reference	Reference	Reference
Mid‐high	1.31 (0.55, 3.13)	0.01 (−0.09, 0.11)	0.05 (−0.05, 0.14)	−0.06 (−0.15, 0.04)	−0.02 (−0.11, 0.08)
Mid‐low	1.14 (0.66, 1.98)	−0.01 (−0.12, 0.11)	0.04 (−0.08, 0.16)	−0.07 (−0.19, 0.04)	0.01 (−0.11, 0.12)
Low	1.31 (0.55, 3.13)	0.13 (−0.06, 0.33)	0.12 (−0.08, 0.31)	0.05 (−0.15, 0.25)	0.10 (−0.10, 0.30)
Paternal educational level					
High	Reference	Reference	Reference	Reference	Reference
Mid‐high	0.65 (0.39, 1.05)	−0.03 (−0.13, 0.07)	−0.05 (−0.15, 0.05)	0.03 (−0.07, 0.15)	0.05 (−0.05, 0.15)
Mid‐low	0.60 (0.34, 1.05)	−0.04 (−0.15, 0.07)	−0.07 (−0.18, 0.04)	0.04 (−0.08, 0.15)	0.04 (−0.08, 0.15)
Low	1.05 (0.54, 2.07)	−0.02 (−0.18, 0.14)	−0.03 (−0.19, 0.13)	0.03 (−0.13, 0.19)	0.01 (−0.15, 0.17)
Net household income					
More than €3200/month	Reference	Reference	Reference	Reference	Reference
€2000‐€3200/month	1.20 (0.74, 1.96)	−0.004 (−0.11, 0.10)	−0.03 (−0.13, 0.08)	0.04 (−0.07, 0.14)	−0.001 (−0.10, 0.10)
Less than €2000/month	1.52 (0.69, 3.34)	**−0.18 (−0.35, −0.003)**	**−0.22 (−0.40, −0.04)**	0.05 (−0.13, 0.23)	−0.01 (−0.19, 0.17)
Financial difficulties					
No	Reference	Reference	Reference	Reference	Reference
Yes	0.90 (0.53, 1.51)	0.09 (−0.02, 0.19)	0.02 (−0.09, 0.13)	0.11 (0.00, 0.22)	0.07 (−0.04, 0.19)
Paternal unemployment					
Paid job	Reference	Reference	Reference	Reference	Reference
No paid job	0.48 (0.16, 1.39)	0.07 (−0.01, 0.15)	0.01 (−0.07, 0.09)	0.10 (0.02, 0.18)	0.14 (0.06, 0.22)
Maternal unemployment					
Paid job	Reference	Reference	Reference	Reference	Reference
No paid job	0.94 (0.56, 1.59)	0.03 (−0.08, 0.14)	0.09 (−0.02, 0.20)	−0.10 (−0.21, 0.01)	−0.08 (−0.19, 0.03)
Ethnic background					
Dutch (n = 3134)	Reference	Reference	Reference	Reference	Reference
Other western (n = 446)	0.93 (0.47, 1.84)	**0.13 (0.001, 0.26)**	0.13 (−0.003, 0.26)	0.02 (−0.11, 0.16)	0.10 (−0.04, 0.23)
Moroccan (n = 267)	1.74 (0.67, 4.55)	0.07 (−0.17, 0.32)	−0.06 (−0.31, 0.18)	0.23 (−0.02, 0.48)	0.16 (−0.09, 0.41)
Turkish (n = 328)	0.44 (0.10, 1.91)	**0.32 (0.12, 0.53)**	0.20 (−0.01, 0.41)	**0.23 (0.02, 0.44)**	**0.37 (0.16, 0.58)**
Surinamese (n = 367)	**2.52 (1.29, 4.90)**	**−0.57 (−0.75, −0.39)**	**−0.71 (−0.89, −0.52)**	**0.23 (0.04, 0.41)**	−0.04 (−0.23, 0.14)
Other nonwestern (n = 678)	1.53 (0.84, 2.78)	−0.03 (−0.17, 0.11)	**−0.20 (−0.35, −0.06)**	**0.29 (0.15, 0.44)**	**0.16 (0.02, 0.31)**

Note

Bold print indicates statistical significance. All sociodemographic factors were added to the model.

^a^ Models were adjusted for maternal age at enrolment, marital status, parity, child's gender and exact age at measurement.

^b^ Models were adjusted for maternal age at enrolment, marital status and parity.

**Table A3:** Associations of socioeconomic status with asthma and lung function at 10 years of age (separate models)^a^

	OR (95% CI)	z Score change (95% CI)			
	Current asthma	FEV_1_	FVC	FEV_1_/FVC	FEF_75_
	n = 4420	n = 4641	n = 4641	n = 4641	n = 4641
Maternal educational level					
High	Reference	Reference	Reference	Reference	Reference
Mid‐high	0.88 (0.60, 1.28)	−0.01 (−0.09, 0.07)	0.03 (−0.05, 0.11)	−0.06 (−0.14, 0.02)	−0.02 (−0.10, 0.06)
Mid‐low	1.00 (0.68, 1.46)	−0.02 (−0.10, 0.07)	−0.01 (−0.09, 0.07)	−0.01 (−0.10, 0.07)	0.05 (−0.04, 0.13)
Low	1.34 (0.83, 2.17)	0.11 (−0.01, 0.22)	0.10 (−0.01, 0.22)	0.02 (−0.10, 0.13)	0.10 (−0.02, 0.21)
Paternal educational level					
High	Reference	Reference	Reference	Reference	Reference
Mid‐high	0.83 (0.55, 1.26)	−0.03 (−0.11, 0.06)	−0.02 (−0.11, 0.06)	−0.002 (−0.09, 0.08)	0.02 (−0.06, 0.11)
Mid‐low	0.82 (0.55, 1.24)	−0.02 (−0.11, 0.06)	−0.05 (−0.14, 0.03)	0.05 (−0.04, 0.13)	0.06 (−0.03, 0.14)
Low	1.06 (0.67, 1.70)	−0.06 (−0.17, 0.04)	−0.08 (−0.18, 0.03)	0.02 (−0.09, 0.12)	0.03 (−0.08, 0.13)
Net household income					
More than €3200/month	Reference	Reference	Reference	Reference	Reference
€2000‐€3200/month	1.31 (0.92, 1.85)	0.03 (−0.05, 0.11)	0.02 (−0.06, 0.09)	0.02 (−0.05, 0.10)	0.04 (−0.04, 0.12)
Less than €2000/month	1.27 (0.79, 2.03)	−0.004 (−0.11, 0.10)	−0.003 (−0.11, 0.10)	−0.01 (−0.11, 0.10)	0.02 (−0.09, 0.13)
Financial difficulties (Yes)	1.19 (0.80, 1.78)	0.09 (−0.001, 0.19)	0.05 (−0.05, 0.14)	0.08 (−0.01, 0.17)	0.09 (−0.01, 0.18)
Paternal unemployment	1.09 (0.58, 2.04)	0.000 (−0.14, 0.14)	0.07 (−0.08, 0.21)	−0.10 (−0.24, 0.04)	−0.08 (−0.22, 0.07)
Maternal unemployment	0.95 (0.66, 1.35)	**0.09 (0.02, 0.17)**	**0.14 (0.06, 0.21)**	−0.07 (−0.15, 0.01)	−0.01 (−0.09, 0.06)

Note

Bold print indicates statistical significance. Models were adjusted for child's ethnic background, gender, exact age at measurement, maternal age at enrolment, marital status and parity.

^a^ Each socioeconomic status indicator was in the model separately.

**Table A4:** Associations of sociodemographic factors with asthma and lung function at 10 years of age (adjustment with additional confounders^a^)

	OR (95% CI)	z Score change (95% CI)			
	Current asthma	FEV_1_	FVC	FEV_1_/FVC	FEF_75_
	n = 4420	n = 4641	n = 4641	n = 4641	n = 4641
Maternal educational level					
High	Reference	Reference	Reference	Reference	Reference
Mid‐high	0.70 (0.35, 1.41)	0.02 (−0.11, 0.15)	0.02 (−0.11, 0.16)	0.004 (−0.13, 0.14)	0.07 (−0.07, 0.20)
Mid‐low	0.98 (0.40, 2.42)	−0.01 (−0.18, 0.16)	0.03 (−0.15, 0.20)	−0.07 (−0.24, 0.11)	0.06 (−0.12, 0.24)
Low	0.57 (0.06, 5.34)	0.25 (−0.19, 0.68)	0.21 (−0.22, 0.65)	0.07 (−0.37, 0.50)	0.23 (−0.21, 0.68)
Paternal educational level					
High	Reference	Reference	Reference	Reference	Reference
Mid‐high	0.54 (0.26, 1.15)	−0.08 (−0.22, 0.06)	−0.07 (−0.21, 0.07)	−0.03 (−0.17, 0.11)	−0.02 (−0.16, 0.12)
Mid‐low	0.38 (0.15, 0.97)	−0.03 (−0.19, 0.13)	−0.01 (−0.17, 0.16)	−0.04 (−0.21, 0.12)	−0.06 (−0.23, 0.11)
Low	0.51 (0.14, 1.80)	−0.09 (−0.35, 0.18)	−0.10 (−0.37, 0.16)	0.01 (−0.26, 0.27)	−0.13 (−0.40, 0.15)
Net household income					
More than €3200/month	Reference	Reference	Reference	Reference	Reference
€2000‐€3200/month	1.84 (0.83, 4.09)	0.06 (−0.10, 0.21)	0.02 (−0.13, 0.18)	0.07 (−0.08, 0.22)	0.05 (−0.11, 0.21)
Less than €2000/month	1.69 (0.37, 7.82)	−0.28 (−0.60, 0.05)	**−0.37 (−0.70, −0.05)**	0.15 (−0.18, 0.47)	0.07 (−0.26, 0.41)
Financial difficulties					
No	Reference	Reference	Reference	Reference	Reference
Yes	0.85 (0.34, 2.12)	−0.004 (−0.19, 0.18)	−0.04 (−0.22, 0.15)	0.05 (−0.13, 0.23)	−0.01 (−0.20, 0.18)
Paternal unemployment					
Paid job	Reference	Reference	Reference	Reference	Reference
No paid job	0.62 (0.08, 5.02)	0.10 (−0.22, 0.43)	0.08 (−0.25, 0.41)	0.04 (−0.29, 0.36)	0.05 (−0.29, 0.39)
Maternal unemployment					
Paid job	Reference	Reference	Reference	Reference	Reference
No paid job	1.29 (0.53, 3.13)	0.10 (−0.09, 0.30)	0.17 (−0.03, 0.37)	−0.12 (−0.31, 0.08)	−0.10 (−0.30, 0.11)
Ethnic background					
Dutch	Reference	Reference	Reference	Reference	Reference
Other western	0.90 (0.33, 2.43)	0.10 (−0.09, 0.29)	0.16 (−0.03, 0.35)	−0.08 (−0.28, 0.11)	0.01 (−0.18, 0.21)
Nonwestern	0.88 (0.36, 2.19)	−0.08 (−0.25, 0.09)	**−0.23 (−0.41, −0.06)**	**0.26 (0.09, 0.44)**	0.15 (−0.03, 0.33)

Note

Bold print indicates statistical significance. All sociodemographic factors were in the model.

^a^ Models were adjusted for child's gender, exact age at measurement, birth weight, gestational age, ever eczema at age 9 years, respiratory tract infections, maternal age at enrolment, marital status, parity, maternal smoking during pregnancy, ever breastfeeding, pets exposure at home, daycare attendance and maternal BMI before pregnancy.

**Table A5:** Associations of sociodemographic factors with lung function in children without current asthma at 10 years of age (N = 3636)

	z Score change (95% CI)			
	FEV_1_	FVC	FEV_1_/FVC	FEF_75_
Maternal educational level				
High	Reference	Reference	Reference	Reference
Mid‐high	0.01 (−0.09, 0.11)	0.07 (−0.03, 0.17)	−0.09 (−0.19, 0.01)	−0.03 (−0.13, 0.07)
Mid‐low	−0.03 (−0.15, 0.09)	0.05 (−0.07, 0.17)	−0.14 (−0.26, −0.01)	−0.03 (−0.15, 0.10)
Low	0.20 (−0.02, 0.42)	0.24 (0.02, 0.46)	−0.05 (−0.27, 0.17)	0.03 (−0.19, 0.25)
Paternal educational level				
High	Reference	Reference	Reference	Reference
Mid‐high	−0.03 (−0.13, 0.08)	−0.05 (−0.16, 0.05)	0.04 (−0.07, 0.14)	0.04 (−0.07, 0.14)
Mid‐low	−0.07 (−0.19, 0.05)	−0.08 (−0.20, 0.04)	−0.01 (−0.12, 0.11)	−0.01 (−0.13, 0.12)
Low	−0.03 (−0.20, 0.14)	−0.03 (−0.19, 0.15)	−0.001 (−0.17, 0.17)	−0.04 (−0.21, 0.13)
Net household income				
More than €3200/month	Reference	Reference	Reference	Reference
€2000‐€3200/month	0.02 (−0.09, 0.13)	−0.01 (−0.12, 0.10)	0.05 (−0.06, 0.15)	0.02 (−0.09, 0.13)
Less than €2000/month	−0.13 (−0.32, 0.06)	**−0.21 (−0.40, −0.01)**	0.12 (−0.07, 0.31)	0.08 (−0.12, 0.27)
Financial difficulties				
No	Reference	Reference	Reference	Reference
Yes	0.14 (0.02, 0.26)	0.07 (−0.05, 0.19)	**0.13 (0.01, 0.24)**	0.10 (−0.02, 0.22)
Paternal unemployment				
Paid job	Reference	Reference	Reference	Reference
No paid job	−0.06 (−0.26, 0.15)	−0.004 (−0.21, 0.20)	−0.09 (−0.30, 0.12)	−0.11 (−0.32, 0.09)
Maternal unemployment				
Paid job	Reference	Reference	Reference	Reference
No paid job	0.03 (−0.08, 0.15)	0.10 (−0.02, 0.22)	**−0.12 (−0.23, −0.001)**	−0.08 (−0.20, 0.04)
Ethnic background				
Dutch	Reference	Reference	Reference	Reference
Other western	0.13 (−0.003, 0.27)	0.13 (−0.002, 0.27)	0.02 (−0.11, 0.16)	0.08 (−0.06, 0.22)
Nonwestern	−0.11 (−0.23, 0.004)	**−0.26 (−0.37, −0.15)**	**0.26 (0.15, 0.38)**	0.13 (0.01, 0.24)

Note

Bold print indicates statistical significance. All sociodemographic factors were in the model. Models were adjusted for maternal age at enrolment, marital status and parity.

**Table A6:** Associations of sociodemographic factors with lung function in children with current asthma at 10 years of age (N = 188)

	z Score change (95% CI)			
	FEV_1_	FVC	FEV_1_/FVC	FEF_75_
Maternal educational level				
High	Reference	Reference	Reference	Reference
Mid‐high	0.15 (−0.50, 0.81)	−0.09 (−0.81, 0.63)	0.40 (−0.37, 1.18)	0.19 (−0.40, 0.79)
Mid‐low	−0.49 (−1.24, 0.26)	**−1.01 (−1.84, −0.18)**	0.83 (−0.06, 1.72)	0.38 (−0.30, 1.05)
Low	−0.89 (−2.16, 0.38)	−1.29 (−2.70, 0.11)	0.71 (−0.82, 2.24)	0.42 (−0.73, 1.58)
Paternal educational level				
High	Reference	Reference	Reference	Reference
Mid‐high	0.03 (−0.61, 0.68)	0.10 (−0.61, 0.81)	−0.14 (−0.90, 0.63)	−0.07 (−0.65, 0.52)
Mid‐low	0.14 (−0.57, 0.85)	0.01 (−0.78, 0.80)	0.21 (−0.64, 1.06)	0.08 (−0.57, 0.72)
Low	0.88 (−0.14, 1.89)	0.59 (−0.54, 1.71)	0.39 (−0.83, 1.62)	0.43 (−0.49, 1.35)
Net household income				
More than €3200/month	Reference	Reference	Reference	Reference
€2000‐€3200/month	0.31 (−0.34, 0.97)	0.52 (−0.21, 1.25)	−0.22 (−1.00, 0.56)	−0.12 (−0.72, 0.47)
Less than €2000/month	0.24 (−0.96, 1.42)	0.48 (−0.83, 1.80)	−0.53 (−2.00, 0.95)	−0.27 (−1.35, 0.81)
Financial difficulties				
No	Reference	Reference	Reference	Reference
Yes	**−0.72 (−1.37, −0.07)**	**−0.72 (−1.44, −0.01)**	0.02 (−0.76, 0.80)	0.002 (−0.59, 0.59)
Paternal unemployment				
Paid job	Reference	Reference	Reference	Reference
No paid job	−**1.63 (−3.25, −0.01)**	−0.78 (−2.56, 1.01)	−1.31 (−3.26, 0.64)	−1.17 (−2.63, 0.30)
Maternal unemployment				
Paid job	Reference	Reference	Reference	Reference
No paid job	0.52 (−0.18, 1.22)	0.45 (−0.32, 1.22)	0.13 (−0.70, 0.95)	0.16 (−0.47, 0.79)
Ethnic background				
Dutch	Reference	Reference	Reference	Reference
Other western	0.10 (−0.87, 1.06)	−0.14 (−1.20, 0.93)	0.33 (−0.84, 1.49)	0.47 (−0.40, 1.35)
Nonwestern	−0.28 (−0.90, 0.34)	−0.41 (−1.09, 0.28)	0.15 (−0.58, 0.88)	−0.10 (−0.65, 0.46)

Note

Bold print indicates statistical significance. All sociodemographic factors were in the model. Models were adjusted for maternal age at enrolment, marital status and parity.
