# Supplementary material for: Sociodemographic factors, current asthma and lung function in an urban child population
Source: Eur J Clin Invest. 2020 Aug 11;50(10):e13277. doi: 10.1111/eci.13277 (PMC7539956; doi:10.1111/eci.13277)
Supplement: Supplementary file 1 — Supplementary Material [file ECI-50-e13277-s001.docx]

Supplementary material. Flowchart of participants included for analysis

N=436

Excluded:

Second child of the same mother: N=427

Third child of the same mother: N=9

N=111

Excluded: missing information on all sociodemographic variables

Children with information on lung function or asthma available

**N=5,237**

FEV_1_ n=4,641

FVC n=4,641

FEV_1_/FVC n=4,641

FEF_75%_  n=4,641

Current asthma n=4,420

Children with information on any sociodemographic variables available

**N=5,673**

Children with information on lung function or asthma available

**N=5,784**

N=1,609

Excluded: missing information on lung function and asthma

Prenatally included children with participation in postnatal phase until 10 years of age

**N=7,393**

**Table A.1. P-values for interaction effects between ethnic background and each socioeconomic status variables on current asthma and lung function measurement**

| **Items** | Current asthma | FEV_1_ | FVC | FEV_1_/FVC | FEF_75_ |
| --- | --- | --- | --- | --- | --- |
|  | **P-value** | **P-value** | **P-value** | **P-value** | **P-value** |
| Ethnic background*maternal educational level | 0.980 | 0.120 | 0.066 | 0.626 | 0.903 |
| Ethnic background*paternal educational level | 0.924 | 0.085 | 0.091 | 0.572 | 0.410 |
| Ethnic background*net household income | 0.596 | 0.471 | 0.514 | 0.089 | 0.553 |
| Ethnic background*financial difficulties | 0.783 | 0.354 | 0.186 | 0.762 | 0.899 |
| Ethnic background*paternal unemployment | 0.958 | 0.483 | 0.920 | 0.332 | 0.492 |
| Ethnic background*maternal unemployment | 0.905 | 0.011 | **0.002** | 0.456 | 0.515 |
| Significant P-values in bold  After applying Bonferroni correction for multiple testing (P=0.10/30=0.003), except interaction effect between ethnic background and maternal unemployment, no statistically significant interaction effect was found. | | | | | |

**Table A.2. Associations of sociodemographic factors with current asthma and lung function at 10 years of age (full models).**

|  | OR (95% CI) | z Score change (95% CI) | | | |
| --- | --- | --- | --- | --- | --- |
|  | Current asthma^*^ | FEV_1_^†^ | FVC^†^ | FEV_1_/FVC^†^ | FEF_75_^†^ |
|  | n=4,420 | n=4,641 | n=4,641 | n=4,641 | n=4,641 |
| Maternal educational level |  |  |  |  |  |
| High | Reference | Reference | Reference | Reference | Reference |
| Mid-high | 1.31 (0.55, 3.13) | 0.01 (-0.09, 0.11) | 0.05 (-0.05, 0.14) | -0.06 (-0.15, 0.04) | -0.02 (-0.11, 0.08) |
| Mid-low | 1.14 (0.66, 1.98) | -0.01 (-0.12, 0.11) | 0.04 (-0.08, 0.16) | -0.07 (-0.19, 0.04) | 0.01 (-0.11, 0.12) |
| Low | 1.31 (0.55, 3.13) | 0.13 (-0.06, 0.33) | 0.12 (-0.08, 0.31) | 0.05 (-0.15, 0.25) | 0.10 (-0.10, 0.30) |
| Paternal educational level |  |  |  |  |  |
| High | Reference | Reference | Reference | Reference | Reference |
| Mid-high | 0.65 (0.39, 1.05) | -0.03 (-0.13, 0.07) | -0.05 (-0.15, 0.05) | 0.03 (-0.07, 0.15) | 0.05 (-0.05, 0.15) |
| Mid-low | 0.60 (0.34, 1.05) | -0.04 (-0.15, 0.07) | -0.07 (-0.18, 0.04) | 0.04 (-0.08, 0.15) | 0.04 (-0.08, 0.15) |
| Low | 1.05 (0.54, 2.07) | -0.02 (-0.18, 0.14) | -0.03 (-0.19, 0.13) | 0.03 (-0.13, 0.19) | 0.01 (-0.15, 0.17) |
| Net household income |  |  |  |  |  |
| More than €3200/month | Reference | Reference | Reference | Reference | Reference |
| €2000-€3200/month | 1.20 (0.74, 1.96) | -0.004 (-0.11, 0.10) | -0.03 (-0.13, 0.08) | 0.04 (-0.07, 0.14) | -0.001 (-0.10, 0.10) |
| Less than €2000/month | 1.52 (0.69, 3.34) | **-0.18 (-0.35, -0.003)** | **-0.22 (-0.40, -0.04)** | 0.05 (-0.13, 0.23) | -0.01 (-0.19, 0.17) |
| Financial difficulties |  |  |  |  |  |
| No | Reference | Reference | Reference | Reference | Reference |
| Yes | 0.90 (0.53, 1.51) | 0.09 (-0.02, 0.19) | 0.02 (-0.09, 0.13) | 0.11 (0.00, 0.22) |  |
| Paternal unemployment |  |  |  |  | 0.07 (-0.04, 0.19) |
| Paid job | Reference | Reference | Reference | Reference | Reference |
| No paid job | 0.48 (0.16, 1.39) | 0.07 (-0.01, 0.15) | 0.01 (-0.07, 0.09) | 0.10 (0.02, 0.18) | 0.14 (0.06, 0.22) |
| Maternal unemployment |  |  |  |  |  |
| Paid job | Reference | Reference | Reference | Reference | Reference |
| No paid job | 0.94 (0.56, 1.59) | 0.03 (-0.08, 0.14) | 0.09 (-0.02, 0.20) | -0.10 (-0.21, 0.01) | -0.08 (-0.19, 0.03) |
| Ethnic background |  |  |  |  |  |
| Dutch (n=3134) | Reference | Reference | Reference | Reference | Reference |
| Other western (n=446) | 0.93 (0.47, 1.84) | **0.13 (0.001, 0.26)** | 0.13 (-0.003, 0.26) | 0.02 (-0.11, 0.16) | 0.10 (-0.04, 0.23) |
| Moroccan (n=267) | 1.74 (0.67, 4.55) | 0.07 (-0.17, 0.32) | -0.06 (-0.31, 0.18) | 0.23 (-0.02, 0.48) | 0.16 (-0.09, 0.41) |
| Turkish (n=328) | 0.44 (0.10, 1.91) | **0.32 (0.12, 0.53)** | 0.20 (-0.01, 0.41) | **0.23 (0.02, 0.44)** | **0.37 (0.16, 0.58)** |
| Surinamese (n=367) | **2.52 (1.29, 4.90)** | **-0.57 (-0.75, -0.39)** | **-0.71 (-0.89, -0.52)** | **0.23 (0.04, 0.41)** | -0.04 (-0.23, 0.14) |
| Other non-western (n=678) | 1.53 (0.84, 2.78) | -0.03 (-0.17, 0.11) | **-0.20 (-0.35, -0.06)** | **0.29 (0.15, 0.44)** | **0.16 (0.02, 0.31)** |

Bold print indicates statistical significance.

All sociodemographic factors were added to the model.

^*^ Models were adjusted for maternal age at enrollment, marital status, parity, child's gender and exact age at measurement.

^†^ Models were adjusted for maternal age at enrollment, marital status and parity.

**Table A.3. Associations of socioeconomic status with asthma and lung function at 10 years of age**

|  | OR (95% CI) | z Score change (95% CI) | | | |
| --- | --- | --- | --- | --- | --- |
|  | Current asthma | FEV_1_ | FVC | FEV_1_/FVC | FEF_75_ |
|  | n=4,420 | n=4,641 | n=4,641 | n=4,641 | n=4,641 |
| Maternal educational level |  |  |  |  |  |
| High | Reference | Reference | Reference | Reference | Reference |
| Mid-high | 0.88 (0.60, 1.28) | -0.01 (-0.09, 0.07) | 0.03 (-0.05, 0.11) | -0.06 (-0.14, 0.02) | -0.02 (-0.10, 0.06) |
| Mid-low | 1.00 (0.68, 1.46) | -0.02 (-0.10, 0.07) | -0.01 (-0.09, 0.07) | -0.01 (-0.10, 0.07) | 0.05 (-0.04, 0.13) |
| Low | 1.34 (0.83, 2.17) | 0.11 (-0.01, 0.22) | 0.10 (-0.01, 0.22) | 0.02 (-0.10, 0.13) | 0.10 (-0.02, 0.21) |
| Paternal educational level |  |  |  |  |  |
| High | Reference | Reference | Reference | Reference | Reference |
| Mid-high | 0.83 (0.55, 1.26) | -0.03 (-0.11, 0.06) | -0.02 (-0.11, 0.06) | -0.002  (-0.09, 0.08) | 0.02 (-0.06, 0.11) |
| Mid-low | 0.82 (0.55, 1.24) | -0.02 (-0.11, 0.06) | -0.05 (-0.14, 0.03) | 0.05 (-0.04, 0.13) | 0.06 (-0.03, 0.14) |
| Low | 1.06 (0.67, 1.70) | -0.06 (-0.17, 0.04) | -0.08 (-0.18, 0.03) | 0.02 (-0.09, 0.12) | 0.03 (-0.08, 0.13) |
| Net household income |  |  |  |  |  |
| More than €3200/month | Reference | Reference | Reference | Reference | Reference |
| €2000-€3200/month | 1.31 (0.92, 1.85) | 0.03 (-0.05, 0.11) | 0.02 (-0.06, 0.09) | 0.02 (-0.05, 0.10) | 0.04 (-0.04, 0.12) |
| Less than €2000/month | 1.27 (0.79, 2.03) | -0.004  (-0.11, 0.10) | -0.003  (-0.11, 0.10) | -0.01 (-0.11, 0.10) | 0.02 (-0.09, 0.13) |
| Financial difficulties (Yes) | 1.19 (0.80, 1.78) | 0.09  (-0.001, 0.19) | 0.05 (-0.05, 0.14) | 0.08 (-0.01, 0.17) | 0.09 (-0.01, 0.18) |
| Paternal unemployment | 1.09 (0.58, 2.04) | 0.000  (-0.14, 0.14) | 0.07 (-0.08, 0.21) | -0.10 (-0.24, 0.04) | -0.08 (-0.22, 0.07) |
| Maternal unemployment | 0.95 (0.66, 1.35) | **0.09 (0.02, 0.17)** | **0.14 (0.06, 0.21)** | -0.07 (-0.15, 0.01) | -0.01 (-0.09, 0.06) |

Bold print indicates statistical significance.

* Each socioeconomic status indicator was in the model separately.

Models were adjusted for child's ethnic background, gender, exact age at measurement, maternal age at enrollment, marital status and parity.

**Table A.4. Associations of sociodemographic factors with asthma and lung function at 10 years of age (adjustment with additional confounders^*^).**

|  | OR (95% CI) | z Score change (95% CI) | | | |
| --- | --- | --- | --- | --- | --- |
|  | Current asthma | FEV_1_ | FVC | FEV_1_/FVC | FEF_75_ |
|  | n=4,420 | n=4,641 | n=4,641 | n=4,641 | n=4,641 |
| Maternal educational level |  |  |  |  |  |
| High | Reference | Reference | Reference | Reference | Reference |
| Mid-high | 0.70 (0.35, 1.41) | 0.02 (-0.11, 0.15) | 0.02 (-0.11, 0.16) | 0.004  (-0.13, 0.14) | 0.07 (-0.07, 0.20) |
| Mid-low | 0.98 (0.40, 2.42) | -0.01 (-0.18, 0.16) | 0.03 (-0.15, 0.20) | -0.07 (-0.24, 0.11) | 0.06 (-0.12, 0.24) |
| Low | 0.57 (0.06, 5.34) | 0.25 (-0.19, 0.68) | 0.21 (-0.22, 0.65) | 0.07 (-0.37, 0.50) | 0.23 (-0.21, 0.68) |
| Paternal educational level |  |  |  |  |  |
| High | Reference | Reference | Reference | Reference | Reference |
| Mid-high | 0.54 (0.26, 1.15) | -0.08 (-0.22, 0.06) | -0.07 (-0.21, 0.07) | -0.03 (-0.17, 0.11) | -0.02 (-0.16, 0.12) |
| Mid-low | 0.38 (0.15, 0.97) | -0.03 (-0.19, 0.13) | -0.01 (-0.17, 0.16) | -0.04 (-0.21, 0.12) | -0.06 (-0.23, 0.11) |
| Low | 0.51 (0.14, 1.80) | -0.09 (-0.35, 0.18) | -0.10 (-0.37, 0.16) | 0.01 (-0.26, 0.27) | -0.13 (-0.40, 0.15) |
| Net household income |  |  |  |  |  |
| More than €3200/month | Reference | Reference | Reference | Reference | Reference |
| €2000-€3200/month | 1.84 (0.83, 4.09) | 0.06 (-0.10, 0.21) | 0.02 (-0.13, 0.18) | 0.07 (-0.08, 0.22) | 0.05 (-0.11, 0.21) |
| Less than €2000/month | 1.69 (0.37, 7.82) | -0.28 (-0.60, 0.05) | **-0.37**  **(-0.70, -0.05)** | 0.15 (-0.18, 0.47) | 0.07 (-0.26, 0.41) |
| Financial difficulties |  |  |  |  |  |
| No | Reference | Reference | Reference | Reference | Reference |
| Yes | 0.85 (0.34, 2.12) | -0.004  (-0.19, 0.18) | -0.04 (-0.22, 0.15) | 0.05 (-0.13, 0.23) | -0.01 (-0.20, 0.18) |
| Paternal unemployment |  |  |  |  |  |
| Paid job | Reference | Reference | Reference | Reference | Reference |
| No paid job | 0.62 (0.08, 5.02) | 0.10 (-0.22, 0.43) | 0.08 (-0.25, 0.41) | 0.04 (-0.29, 0.36) | 0.05 (-0.29, 0.39) |
| Maternal unemployment |  |  |  |  |  |
| Paid job | Reference | Reference | Reference | Reference | Reference |
| No paid job | 1.29 (0.53, 3.13) | 0.10 (-0.09, 0.30) | 0.17 (-0.03, 0.37) | -0.12 (-0.31, 0.08) | -0.10 (-0.30, 0.11) |
| Ethnic background |  |  |  |  |  |
| Dutch | Reference | Reference | Reference | Reference | Reference |
| Other western | 0.90 (0.33, 2.43) | 0.10 (-0.09, 0.29) | 0.16 (-0.03, 0.35) | -0.08 (-0.28, 0.11) | 0.01 (-0.18, 0.21) |
| Non-western | 0.88 (0.36, 2.19) | -0.08 (-0.25, 0.09) | **-0.23**  **(-0.41, -0.06)** | **0.26 (0.09, 0.44)** | 0.15 (-0.03, 0.33) |

Bold print indicates statistical significance.

All sociodemographic factors were in the model.

* Models were adjusted for child's gender, exact age at measurement, birth weight, gestational age, ever eczema at age 9 years, respiratory tract infections, maternal age at enrollment, marital status, parity, maternal smoking during pregnancy, ever breastfeeding, pets exposure at home, daycare attendance and maternal BMI before pregnancy.

**Table A.5. Associations of sociodemographic factors with lung function in children without current asthma at 10 years of age (N=3,636).**

|  | z Score change (95% CI) | | | |
| --- | --- | --- | --- | --- |
|  | FEV_1_ | FVC | FEV_1_/FVC | FEF_75_ |
| Maternal educational level |  |  |  |  |
| High | Reference | Reference | Reference | Reference |
| Mid-high | 0.01 (-0.09, 0.11) | 0.07 (-0.03, 0.17) | -0.09 (-0.19, 0.01) | -0.03 (-0.13, 0.07) |
| Mid-low | -0.03 (-0.15, 0.09) | 0.05 (-0.07, 0.17) | -0.14 (-0.26, -0.01) | -0.03 (-0.15, 0.10) |
| Low | 0.20 (-0.02, 0.42) | 0.24 (0.02, 0.46) | -0.05 (-0.27, 0.17) | 0.03 (-0.19, 0.25) |
| Paternal educational level |  |  |  |  |
| High | Reference | Reference | Reference | Reference |
| Mid-high | -0.03 (-0.13, 0.08) | -0.05 (-0.16, 0.05) | 0.04 (-0.07, 0.14) | 0.04 (-0.07, 0.14) |
| Mid-low | -0.07 (-0.19, 0.05) | -0.08 (-0.20, 0.04) | -0.01 (-0.12, 0.11) | -0.01 (-0.13, 0.12) |
| Low | -0.03 (-0.20, 0.14) | -0.03 (-0.19, 0.15) | -0.001 (-0.17, 0.17) | -0.04 (-0.21, 0.13) |
| Net household income |  |  |  |  |
| More than €3200/month | Reference | Reference | Reference | Reference |
| €2000-€3200/month | 0.02 (-0.09, 0.13) | -0.01 (-0.12, 0.10) | 0.05 (-0.06, 0.15) | 0.02 (-0.09, 0.13) |
| Less than €2000/month | -0.13 (-0.32, 0.06) | **-0.21 (-0.40, -0.01)** | 0.12 (-0.07, 0.31) | 0.08 (-0.12, 0.27) |
| Financial difficulties |  |  |  |  |
| No | Reference | Reference | Reference | Reference |
| Yes | 0.14 (0.02, 0.26) | 0.07 (-0.05, 0.19) | **0.13 (0.01, 0.24)** | 0.10 (-0.02, 0.22) |
| Paternal unemployment |  |  |  |  |
| Paid job | Reference | Reference | Reference | Reference |
| No paid job | -0.06 (-0.26, 0.15) | -0.004 (-0.21, 0.20) | -0.09 (-0.30, 0.12) | -0.11 (-0.32, 0.09) |
| Maternal unemployment |  |  |  |  |
| Paid job | Reference | Reference | Reference | Reference |
| No paid job | 0.03 (-0.08, 0.15) | 0.10 (-0.02, 0.22) | **-0.12 (-0.23, -0.001)** | -0.08 (-0.20, 0.04) |
| Ethnic background |  |  |  |  |
| Dutch | Reference | Reference | Reference | Reference |
| Other western | 0.13 (-0.003, 0.27) | 0.13 (-0.002, 0.27) | 0.02 (-0.11, 0.16) | 0.08 (-0.06, 0.22) |
| Non-western | -0.11 (-0.23, 0.004) | **-0.26 (-0.37, -0.15)** | **0.26 (0.15, 0.38)** | 0.13 (0.01, 0.24) |

Bold print indicates statistical significance.

All sociodemographic factors were in the model.

Models were adjusted for maternal age at enrollment, marital status and parity.

**Table A.6. Associations of sociodemographic factors with lung function in children with current asthma at 10 years of age (N=188).**

|  | z Score change (95% CI) | | | |
| --- | --- | --- | --- | --- |
|  | FEV_1_ | FVC | FEV_1_/FVC | FEF_75_ |
| Maternal educational level |  |  |  |  |
| High | Reference | Reference | Reference | Reference |
| Mid-high | 0.15 (-0.50, 0.81) | -0.09 (-0.81, 0.63) | 0.40 (-0.37, 1.18) | 0.19 (-0.40, 0.79) |
| Mid-low | -0.49 (-1.24, 0.26) | **-1.01 (-1.84, -0.18)** | 0.83 (-0.06, 1.72) | 0.38 (-0.30, 1.05) |
| Low | -0.89 (-2.16, 0.38) | -1.29 (-2.70, 0.11) | 0.71 (-0.82, 2.24) | 0.42 (-0.73, 1.58) |
| Paternal educational level |  |  |  |  |
| High | Reference | Reference | Reference | Reference |
| Mid-high | 0.03 (-0.61, 0.68) | 0.10 (-0.61, 0.81) | -0.14 (-0.90, 0.63) | -0.07 (-0.65, 0.52) |
| Mid-low | 0.14 (-0.57, 0.85) | 0.01 (-0.78, 0.80) | 0.21 (-0.64, 1.06) | 0.08 (-0.57, 0.72) |
| Low | 0.88 (-0.14, 1.89) | 0.59 (-0.54, 1.71) | 0.39 (-0.83, 1.62) | 0.43 (-0.49, 1.35) |
| Net household income |  |  |  |  |
| More than €3200/month | Reference | Reference | Reference | Reference |
| €2000-€3200/month | 0.31 (-0.34, 0.97) | 0.52 (-0.21, 1.25) | -0.22 (-1.00, 0.56) | -0.12 (-0.72, 0.47) |
| Less than €2000/month | 0.24 (-0.96, 1.42) | 0.48 (-0.83, 1.80) | -0.53 (-2.00, 0.95) | -0.27 (-1.35, 0.81) |
| Financial difficulties |  |  |  |  |
| No | Reference | Reference | Reference | Reference |
| Yes | **-0.72 (-1.37, -0.07)** | **-0.72 (-1.44, -0.01)** | 0.02 (-0.76, 0.80) | 0.002 (-0.59, 0.59) |
| Paternal unemployment |  |  |  |  |
| Paid job | Reference | Reference | Reference | Reference |
| No paid job | **-1.63 (-3.25, -0.01)** | -0.78 (-2.56, 1.01) | -1.31 (-3.26, 0.64) | -1.17 (-2.63, 0.30) |
| Maternal unemployment |  |  |  |  |
| Paid job | Reference | Reference | Reference | Reference |
| No paid job | 0.52 (-0.18, 1.22) | 0.45 (-0.32, 1.22) | 0.13 (-0.70, 0.95) | 0.16 (-0.47, 0.79) |
| Ethnic background |  |  |  |  |
| Dutch | Reference | Reference | Reference | Reference |
| Other western | 0.10 (-0.87, 1.06) | -0.14 (-1.20, 0.93) | 0.33 (-0.84, 1.49) | 0.47 (-0.40, 1.35) |
| Non-western | -0.28 (-0.90, 0.34) | -0.41 (-1.09, 0.28) | 0.15 (-0.58, 0.88) | -0.10 (-0.65, 0.46) |

Bold print indicates statistical significance.

All sociodemographic factors were in the model.

Models were adjusted for maternal age at enrollment, marital status and parity.
